# Combining Mechanisms of Growth Arrest in Solid Tumours: A Mathematical Investigation

**DOI:** 10.1007/s11538-022-01034-2

**Published:** 2022-07-01

**Authors:** Chloé Colson, Helen M. Byrne, Philip K. Maini

**Affiliations:** Wolfson Centre for Mathematical Biology, Mathematical Institute, University of Oxford, Radcliffe Observatory Quarter, Oxford, OX2 6GG UK

**Keywords:** Tumour growth, Growth-limiting mechanisms, Ordinary differential equation model, Bi-stability

## Abstract

The processes underpinning solid tumour growth involve the interactions between various healthy and tumour tissue components and the vasculature, and can be affected in different ways by cancer treatment. In particular, the growth-limiting mechanisms at play may influence tumour responses to treatment. In this paper, we propose a simple ordinary differential equation model of solid tumour growth to investigate how tumour-specific mechanisms of growth arrest may affect tumour response to different combination cancer therapies. We consider the interactions of tumour cells with the physical space in which they proliferate and a nutrient supplied by the tumour vasculature, with the aim of representing two distinct growth arrest mechanisms. More specifically, we wish to consider growth arrest due to (1) nutrient deficiency, which corresponds to balancing cell proliferation and death rates, and (2) competition for space, which corresponds to cessation of proliferation without cell death. We perform numerical simulations of the model and a steady-state analysis to determine the possible tumour growth scenarios described by the model. We find that there are three distinct growth regimes: the nutrient- and spatially limited regimes and a bi-stable regime, in which both growth arrest mechanisms are simultaneously active. Thus, the proposed model has the features required to investigate and distinguish tumour responses to different cancer treatments.

**Supplementary Information:**

The online version contains supplementary material available at 10.1007/s11538-022-01034-2.

## Introduction

Solid tumour growth is a complex process involving interactions between multiple different cell types, e.g. healthy, tumour and immune cells, as well as other tissue components such as the extra-cellular matrix and the vasculature. Each of these, and their interactions, can be affected in different ways by anti-cancer therapies, which has prompted extensive experimental and theoretical research on tumour growth dynamics. In particular, many mathematical models, of varying complexity, have been proposed to describe tumour growth. These models typically include a single, or a grouping of, growth-limiting process(es) which can significantly influence treatment response. In this paper, we propose an ordinary differential equation (ODE) model of tumour growth that distinguishes between two alternative mechanisms for growth arrest of a tumour population. In this way, our simple model lends itself to future studies of the effectiveness of different combination cancer therapies.

*Tumour growth dynamics.* Tumour growth is typically separated into two stages: the avascular and the vascular stages (Chaplain 1996). During the avascular phase, all tumour cells initially proliferate by consuming nutrients that diffuse from blood vessels in neighbouring healthy tissue, resulting in an initial exponential growth of the tumour. As the tumour’s size increases and cell numbers grow, the outermost cells begin to form a proliferative rim while those in the central regions gradually become quiescent (i.e. alive, but non-proliferative) as their access to crucial nutrients is cut off. This results in linear growth of the tumour. Quiescent cells eventually die due to prolonged nutrient deprivation, forming a necrotic core within the tumour that decays at a certain rate. Thus, an avascular tumour gradually approaches a diffusion-limited, equilibrium size as the rate of growth due to cell proliferation in the well-oxygenated outer rim balances with the rate of cell degradation in the nutrient-starved necrotic region. In order to progress, the tumour must develop its own vasculature via angiogenesis (Chaplain 1996), which triggers the growth of new blood vessels towards the tumour. Once vascularised, a tumour can access the nutrients necessary for further, rapid growth and discard metabolic waste. Blood vessels additionally provide a means for tumour cells to travel to other tissues, where they may establish secondary tumours or metastases.

*Mathematical models of tumour growth and their growth-limiting mechanisms.* Both stages of tumour growth have been modelled, either independently or in combination, using a range of approaches. This includes phenomenological ODE models as well as more detailed, spatially resolved models such as partial differential equation (PDE) models, discrete, individual-based models (IBMs) and hybrid models combining differential equations and IBMs. For detailed reviews of spatially averaged continuum models, see Murphy et al. (2016), and, of spatially resolved continuum, discrete and hybrid models of tumour growth, see Araujo and McElwain (2004), Byrne (2012), Roose et al. (2007), Cristini and Lowengrub (2010), Martins et al. (2007), and Deisboeck et al. (2011), respectively.

A common characteristic of most of these models is their focus on describing either a single growth-limiting process or a group of growth-limiting processes, whose effects cannot be distinguished. For example, some phenomenological ODE models (e.g. Gompertz, logistic, etc. (Murphy et al. 2016)) predict the growth of an avascular tumour to a constant (in time) limiting size, called the carrying capacity. The latter can be defined in different ways: the available free space for the tumour to occupy (Liu et al. 2021), the volume that can be reached given nutrient availability (Zahid et al. 2021), among others (Milzman et al. 2021). Regardless of the precise definition of this carrying capacity, a single mechanism for growth arrest of the tumour is explicitly considered: a tumour reaches steady state as the proportion of proliferating cells converges to zero (with no explicit cell death). This also holds for ODE models that incorporate a time-dependent carrying capacity, e.g. the model of vascular tumour growth developed by Hahnfeldt et al. (1999), which assumes a time-dependent carrying capacity to account for dynamic changes in the vascular support available to the tumour.

In terms of spatially resolved models, the seminal model developed by Greenspan (1972) describes diffusion-limited (avascular) tumour growth, where the tumour reaches an equilibrium size as the death of cells in nutrient-poor regions balances the birth of cells in nutrient-rich regions. This same single growth arrest mechanism is also described by the multiphase model of avascular tumour growth developed by Lewin et al. (2020). Further, the model of vascular tumour growth developed by Panovska et al. (2007), which comprises a coupled system of nonlinear PDEs, also depicts a tumour that reaches an equilibrium size as cell death and cell birth balance one another. However, they consider mechanical constraints to tumour growth (which limit cell proliferation) in addition to those imposed by nutrient availability (which lead to cell death), i.e. they incorporate several growth-limiting processes, but their effects are combined from a mathematical perspective. In contrast to the aforementioned spatially resolved, continuum models, Enderling et al. (2006) extend the model of tumour invasion developed by Anderson (2005) to represent a tumour which attains an equilibrium size as the remaining available space for tumour cells decreases to zero. In particular, they predict growth arrest due to a cessation of proliferation, with no cell death. As a final example, Drasdo and Höhme (2005) developed a discrete model of *in vitro* tumour growth which describes the growth of monolayers and multicellular spheroids. The key growth-limiting factor for monolayers is contact inhibition, whereas it is nutrient deficiency for spheroids. Despite the differences between these two growth-limiting processes, they both can lead to a tumour reaching an equilibrium size due to the balance of cell proliferation and cell death.

The above brief and non-exhaustive summary illustrates that a significant amount of research has focused on developing models of solid tumour growth of increasing biological complexity. Yet, they all typically share the common feature of describing a single mechanism by which a tumour population may reach a steady state, i.e. via cessation of cell proliferation without any cell death or via the balancing of cell proliferation and cell death. The question of how to strike the balance between the biological detail of a mathematical model, which constrains it, and its suitability for making clinically relevant predictions (e.g. through data fitting and parameter estimation) therefore poses itself.

*Structure of the paper.* In the present paper, we aim to derive a model of vascular tumour growth that retains the simplicity of phenomenological models, while providing additional mechanistic insight and capturing the key behaviour of more complex models. We focus on vascular tumours as, in practice, tumours are likely to have reached this stage by the time they are detected. Further, the effect of various treatments on the vasculature can be significant, especially when considering combination treatments. A main feature we seek for our model is that it can distinguish between two alternative growth-limiting mechanisms:

1. growth arrest in response to nutrient deficiency, which translates into the balancing of cell proliferation and death,
2. growth arrest in response to competition for space, which translates into a cessation of all proliferation, with no cell death.

As a result, the model provides a means to understand how mechanisms of growth limitation may affect tumour responses to treatment, particularly when both mechanisms are simultaneously active.

This paper is structured as follows. In Sect. 2, we derive a new ODE model of vascular tumour growth. We investigate the model behaviour in Sect. 3 by performing numerical simulations and a steady-state analysis. The paper concludes in Sect. 4, where we discuss our findings and avenues for future work.

## The Mathematical Model

In this section, we present a new model of vascular tumour growth, which considers the interactions of a tumour with the physical space in which it is growing and a nutrient supplied by the tumour vasculature. Our first key simplifying assumption is that the vascular volume remains constant during tumour growth. In particular, we neglect angiogenesis and vascular remodelling and view the vascular volume, *V*, as a model parameter (rather than a dynamic variable) which influences the availability of nutrient and space and, thus, the tumour’s carrying capacity. We make this simplifying assumption in order to limit the complexity of the ODE model we are proposing, which enables us to focus on our aim of describing two different growth-limiting mechanisms using a model that exhibits rich solution structure despite its simplicity. We will come back to this point in the Discussion.

Now, denoting the tumour cell volume by *T* and the nutrient concentration by *c*, we propose the following system of time-dependent ODEs:

1 $$\begin{aligned}&\frac{\hbox {d}T}{\hbox {d}t} = \underbrace{q^*_2cT(S_{\max } - (T+V))}_{\begin{array}{c} {\text {rate of}} \\ {\text {tumour cell proliferation}} \end{array}} - \underbrace{\left[ \delta ^*_1(c^*_{\min } - c)\right] H(c^*_{\min }-c)T}_{\begin{array}{c} {\text {rate of}} \\ {\text {cell death due to nutrient starvation}} \end{array}}, \end{aligned}$$

2 $$\begin{aligned}&\frac{\hbox {d}c}{\hbox {d}t} = \underbrace{g^*(c^*_{\max } -c)V}_{\begin{array}{c} {\text {rate of}} \\ {\text {nutrient delivery}} \end{array}} - \underbrace{q^*_1cT}_{\begin{array}{c} {\text {baseline rate of}} \\ {\text {nutrient consumption} } \end{array}}- \underbrace{q^*_3cT(S_{\max } - (T+V))}_{\begin{array}{c} {\text {additional rate of}} \\ {\text {nutrient consumption for proliferation}} \end{array}}, \end{aligned}$$

where

3 $$\begin{aligned} V \equiv V^*_0 {:}{=}V(0) \quad \text {and} \quad H(x) = {\left\{ \begin{array}{ll} 1, \,\, \text {if } x \ge 0, \\ 0, \,\, \text {if } x < 0. \end{array}\right. } \end{aligned}$$

Our model is based on the following assumptions:

- There is a fixed amount of physical space, $S_{\max }$, which can be occupied by *T* and *V*; the available free space is $S = S_{\max } - (T+V)$.
- Tumour cells, *T*, need space and nutrient in order to proliferate. We assume that they proliferate at a rate proportional to the nutrient concentration, *c*, and the amount of free space available, *S*, with constant of proportionality $q^*_2 > 0$.
- Below a fixed nutrient concentration, $c^*_{\min }$, satisfying $0< c^*_{\min } < c^*_{\max }$, tumour cells die of nutrient starvation at a rate that is a monotonically decreasing function of *c* for $0 \le c < c^*_{\min }$ such that (i) no tumour cells die at $c=c^*_{\min }$ and (ii) when $c=0$ the death rate of tumour cells attains its maximal value of $\delta ^*_1 c^*_{\min }$, where $\delta ^*_1 > 0$ is a constant of proportionality. We refer to $c^*_{\min }$ as the threshold for severe hypoxia, which is defined as a state of oxygen deficiency.
- A nutrient, *c*, chosen to be oxygen, is supplied by the tumour vasculature, *V*, at a rate that is a monotonically decreasing function of *c* for $0 \le c \le c^*_{\max }$ such that (i) no oxygen is supplied once the oxygen concentration in the tumour attains the value $c^*_{\max } > 0$ and (ii) the maximal supply rate of oxygen is attained when $c=0$ and is equal to $g^* c^*_{\max }$, with $g^* > 0$. Here, $c_{\max }^*$ represents the oxygen concentration in the vasculature (note that, from (2), ${c}\le {c}^{*}_{\text {max}}$) and $g^*$ represents the rate of oxygen exchange per unit volume area of blood vessels.
- Tumour cells consume *c* for maintenance at a rate proportional to *c*, with rate constant $q^*_1 > 0$. They also consume *c* for proliferation at a rate proportional to the proliferation rate, with a conversion factor $k^* > 0$ defined such that $q^*_3 = \frac{q^*_2}{k^*} >0$.

In order to reduce the number of parameters in our model, we non-dimensionalise the system (1)–(3) by introducing the following scalings:

$$\begin{aligned} \widehat{T} = \frac{T}{S_{\max }}, \quad \widehat{S} = \frac{S}{S_{\max }}, \quad \widehat{V} = \frac{V}{S_{\max }}, \quad \widehat{c}= \frac{c}{c^*_{\max }}, \quad \widehat{t} = \tau t. \end{aligned}$$

Recalling that $V \equiv V^*_0$ and dropping hats for notational convenience, we obtain the following system:

4 $$\begin{aligned}&\frac{\hbox {d}T}{\hbox {d}t} = {q_2}cT(1 - (T+V_0)) - \delta _1(c_{\min } - c)H(c_{\min }-c)T \end{aligned}$$

5 $$\begin{aligned}&{\frac{\hbox {d}c}{\hbox {d}t} =g(1-c)V_0 - q_1cT - q_3cT(1- (T+V_0)),} \end{aligned}$$

where

6 $$\begin{aligned} \begin{aligned} V_0&= \frac{V_0^*}{S_{\max }}, \quad {q_1}= \frac{q^*_1 S_{\max }}{\tau }, \quad {q_3}= \frac{q^*_3 S_{\max }^2}{\tau }, \quad q_2 = \frac{q^*_2 S_{\max } c^*_{\max }}{\tau }, \\ c_{\min }&= \frac{c^*_{\min }}{c^*_{\max }}, \quad \delta _1= \frac{\delta ^*_1 c^*_{\max }}{\tau }, \quad g = \frac{g^* S_{\max }}{\tau }. \end{aligned} \end{aligned}$$

Given that we defined $k^* >0$ such that $q^*_3 = \frac{q^*_2}{k^*}$, we can define $k > 0$ such that $q_3 = \frac{q_2}{k}$, where

7 $$\begin{aligned} k = \frac{c^*_{\max }}{S_{\max }}k^*. \end{aligned}$$

## Investigating Model Behaviour

### Numerical Simulations: Tumour Growth Curves

In this section, we present numerical simulations of the tumour growth model (4)–(5).

*Numerical setup.* We solve the ODE system (4)–(5) for $t \in (0, T]$, where $T > 0$, using ODE45, a single-step MATLAB built-in solver for non-stiff ODEs that is based on an explicit Runge–Kutta (4,5) formula, the Dormand–Prince pair. We impose the initial conditions $(T(0),c(0))= (0.05,1)$. We choose $T(0)=0.05$ so that the tumour is initially much smaller than the available free space (i.e. $0 < T \ll 1$), but also large enough to be vascular with $V_0 \in (0,0.005]$. The choice of $c(0)=1$ is so that we consider tumours which are, initially, well oxygenated.

The focus of this work is on characterising the qualitative behaviour of the model and assessing its suitability for benchmarking combination cancer treatments. As such, accurately estimating parameter values by fitting the model to experimental data is beyond the scope of this work. We instead combine parameter values from different sources in the literature and preliminary numerical simulations to fix the values of $c_{\min }$, *g* and *k*. Further, we consider a biologically realistic range of possible values for the remaining parameters. Our dimensionless parameter choices, listed in Table 1, are motivated by the arguments included in “Appendix A1”.

**Table 1:** List of dimensionless parameters and values assigned to them for the purpose of the model analysis and numerical simulations

Parameter	Definition	Value(s)
$c_{\min }$	Anoxic oxygen threshold	$10^{-2}$
*g*	Rate of oxygen exchange per unit vascular volume	5
*k*	Conversation factor	$10^{-2}$
$$q_1$$	$$O_2$$ consumption rate for maintenance	$$ [10^{-2}, 10]$$
$$q_3$$	$$O_2$$ consumption rate for proliferation	$$ [10^{-2}, 10]$$
$$q_2$$	Proliferation rate	$$ q_3/k$$
$$\delta _1$$	Rate of death by nutrient starvation	$$q_2 $$
$V_0$	Initial vascular volume	$(0,5 \times 10^{-3}]$

#### Remark 1

A key simplifying assumption we make in defining the model parameters is that the dimensionless rate of cell death due to nutrient starvation, $\delta _1$, is equal to the dimensionless rate of cell proliferation, $q_2$. This enables us to reduce the number of system parameters, making it more tractable. We motivate this assumption with experimental evidence which suggests that tumour cell proliferation and death rates can be highly correlated for some tumours (Leoncini et al. 1993; Liu et al. 2001; Vaquero et al. 2004). This would suggest that these parameters are proportional to each other. Our numerical and analytical studies reveal that the key results in this paper are not affected by the value of this constant of proportionality (results not shown) and we, therefore, set it equal to 1.

**Fig. 1:**
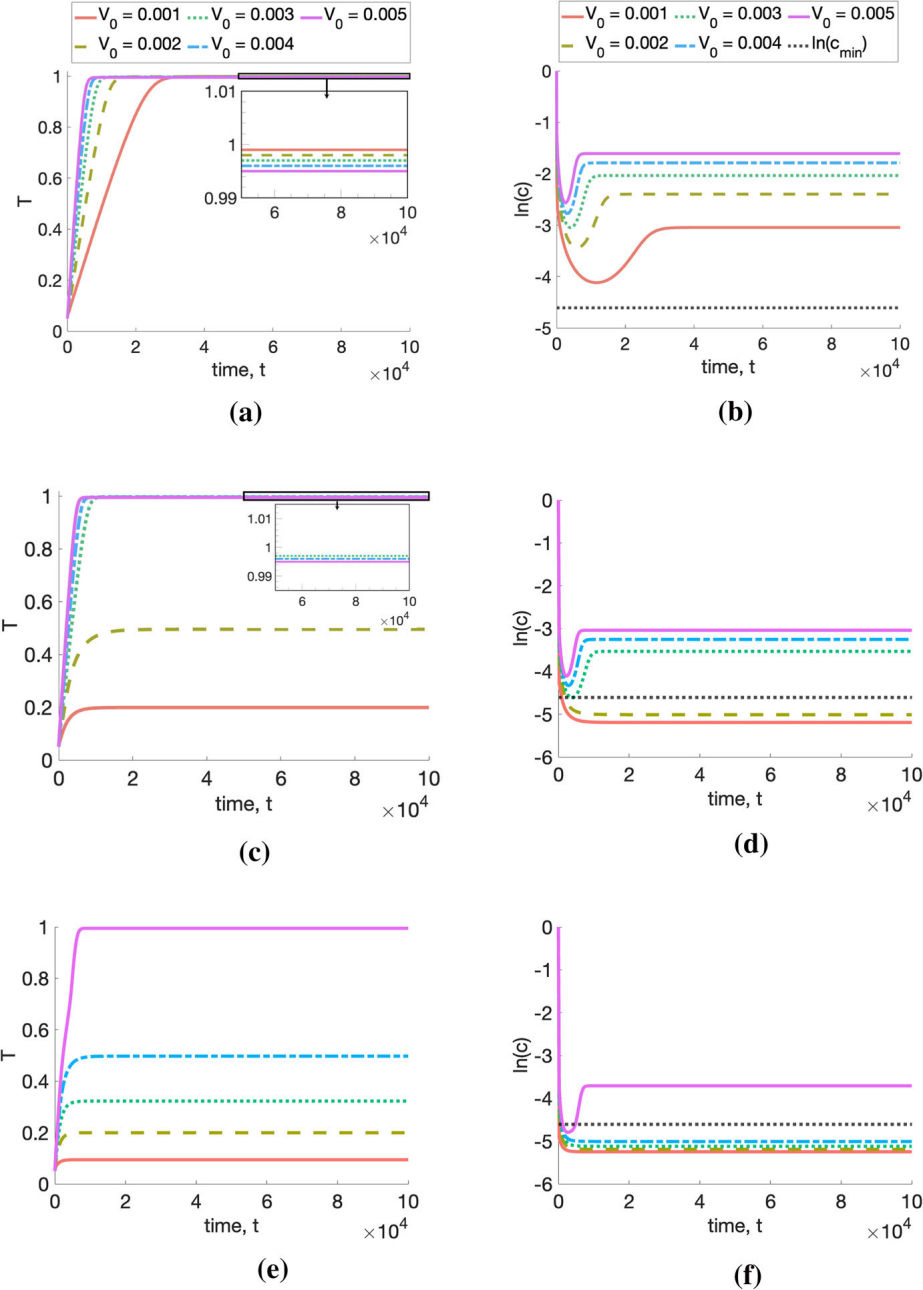
We solve Eqs. (4)–(5) for $t \in (0,10^5]$ subject to the initial conditions $(T(0),c(0)) = (0.05, 1)$. In plots (**a**, **b**), (**c**, **d**) and (**e**, **f**), we fix $(q_1,q_3)$ = (0.1, 1), $(q_1,q_3)$ = (0.5, 5) and $(q_1,q_3)$ = (1, 10), respectively, and vary $V_0 \in \{0.001, 0.002, 0.003, 0.004, 0.005 \}$. In (**a**, **c**, **e**), we plot the time evolution of the tumour volume, *T*(*t*); in (**b**, **d**, **f**), we depict the evolution of the logarithm of the oxygen concentration, *c*(*t*). In (**a**) and (**c**), we also include a magnified view of the growth curves as they approach their respective steady states. All tumours grow to a limiting size, which appears to be a decreasing function of $q_1$ and $q_3$. Further, the tumour steady state appears to be an increasing function of $V_0$ when the steady-state oxygen concentration is below $c_{\min }$, and a decreasing function of $V_0$ otherwise. In contrast, the steady-state oxygen concentration always increases with $V_0$

*Simulating tumour growth.* For fixed values of $q_1$ and $q_3$, we observe how tumour growth dynamics change as we vary the initial vascular volume, $V_0$. The results presented in Fig. 1 show that the model exhibits initial exponential or linear growth followed by a growth slow down as the tumour reaches a limiting size. These dynamics are characteristic of both experimental data and existing phenomenological models (Drasdo and Höhme 2005; Koziol et al. 2020; Murphy et al. 2016).

We note also that the tumour-specific parameters $q_1$, $q_3$ and $V_0$ appear to determine the tumour’s limiting size, which can vary quite significantly from one parameter set to another (Figs. 1c, e). In particular, studying Fig. 1d, f, which represent the evolution of the logarithm of the oxygen concentration in the tumour, *c*, alongside Fig. 1c, e, suggests that the large differences between tumour limiting sizes arise as a result of whether *c* is above or below the oxygen threshold for severe hypoxia, $c_{\min }$. This hints at the existence of different growth regimes, which could be characterised by different growth-limiting mechanisms. We investigate this further by performing a steady-state analysis, which is presented in the following section.

### Steady-State Analysis

We now perform a steady-state analysis for the system (4)–(5) in order to understand how varying $q_1, q_3$ and $V_0$, the oxygen consumption rates for proliferation and maintenance and the initial vascular volume, respectively, impacts the equilibrium tumour volume attained in the long term. The model dynamics are different depending on whether we are in a nutrient-rich regime, i.e. $c_{\min } \le c \le 1$, or in a nutrient-poor regime, i.e. $0 \le c < c_{\min }$. We therefore investigate the existence and stability of steady-state solutions in each of these regimes separately, referring to steady states satisfying $c_{\min } \le c \le 1$ as spatially limited (SL), while steady states satisfying $0 \le c < c_{\min }$ are called nutrient-limited (NL).

#### Steady-State Solutions: Existence and Multiplicity

*Spatially Limited Steady States.* In this case, the nutrient is plentiful (i.e. $c_{\min } \le c \le 1)$ and we find the steady-state solutions by solving: 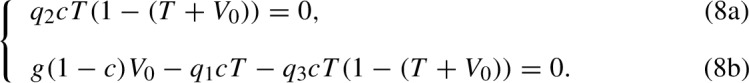


Since $0< V_0 < 1$, it is straightforward to show that there are two steady states:

9 $$\begin{aligned} \mathrm {SS}_1:&\quad (T_1^*,c_1^*) = (0,1), \end{aligned}$$

10 $$\begin{aligned} \mathrm {SS}_2:&\quad (T_2^*,c_2^*) = \left( 1 - V_0, \frac{V_0}{V_0 + (q_1/g) (1-V_0)}\right) . \end{aligned}$$

Clearly, $\mathrm {SS}_1$ is an admissible steady state, and the condition on $V_0$ implies that $T_2^*=1-V_0 > 0$ for $\mathrm {SS}_2$. For $c_2^*$ to lie in the appropriate nutrient regime, we require $c_2^* \ge c_{\min }$ (as $c_2^* < 1$ follows trivially). This condition is satisfied when the following relationship holds:

11 $$\begin{aligned} V_0\ge \frac{1}{\frac{g}{q_1} \frac{(1-c_{\min })}{c_{\min }}+1}. \end{aligned}$$

Now, steady state $\mathrm {SS}_1$ represents a tumour-free state, which exists for all combinations of parameters. In contrast, steady state $\mathrm {SS}_2$ represents tumours which occupy all of the available free space; their growth is limited by space availability, and at steady state, there is neither cell proliferation nor death. For *g* and $c_{\min }$ fixed, the inequality (11) implies that $\mathrm {SS}_2$ exists if $V_0$ is bounded below by an increasing function of $q_1$. In other words, if the tumour vasculature can support the tumour’s maintenance needs at equilibrium, then $\mathrm {SS}_2$ exists.

*Nutrient-Limited Steady States.* In this case, there is a nutrient shortage (i.e. $0 \le c < c_{\min }$) and we determine the steady-state values by solving: 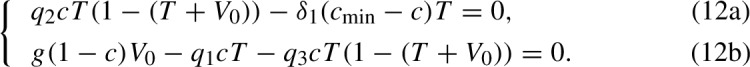 The trivial steady state for *T* implies that, as $V_0>0$, the steady state for *c* must be 1. However, this is not a valid steady state in the NL regime, since ${ 1 = c > c_{\min }}$. Therefore, we suppose $T>0$ and find solutions of (12a)–(12b) by determining the intersection points of the *T*- and *c*-nullclines. Using (12a), we find that the nonzero *T*-nullcline is given by:

13 $$\begin{aligned} T(c) = (1-V_0)- \frac{\delta _1}{q_2}\left( \frac{c_{\min }}{c} -1\right) , \end{aligned}$$

which, given that we assume $\delta _1 = q_2$, reduces to

14 $$\begin{aligned} T(c) = (1 - V_0) - \left( \frac{c_{\min }}{c} -1\right) . \end{aligned}$$

To determine the *c*-nullcline, we solve the quadratic equation (12b) for *T* and find it has two branches:

15 $$\begin{aligned} T_{\pm }(c) = \frac{q_1 + q_3(1-V_0) \pm \sqrt{(q_1 + q_3(1-V_0))^2 - 4 q_3 g V_0 (1-c)/c}}{2q_3}. \end{aligned}$$

We can now determine the steady-state values for *c* by equating (14) and each branch in (15) and solving for $c_\pm$ corresponding to $T_\pm$. We find:
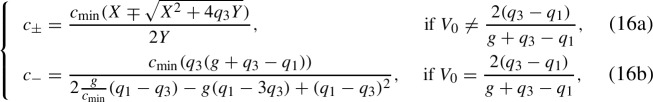
 where

17 $$\begin{aligned} {\left\{ \begin{array}{ll} X = q_1-3q_3+\left( \frac{g}{c_{\min }}+q_3\right) V_0,\\ Y = 2(q_1-q_3)+(g+q_3 -q_1)V_0. \end{array}\right. } \end{aligned}$$

We note here that we obtain a unique steady-state value for *c* (i.e. (16b)) when the curve *T*(*c*) defined by (14) intersects only one of the branches $T_\pm (c)$ defined by (15). In particular, *T*(*c*) intersects $T_-(c)$.

Finally, this implies that there are up to two NL steady states:

18 $$\begin{aligned} \mathrm {SS}_3:&\quad (T_3^*,c_3^*) = \left( T(c_-), c_-\right) \end{aligned}$$

19 $$\begin{aligned} \mathrm {SS}_4:&\quad (T_4^*,c_4^*) = \left( T(c_+), c_+\right) , \quad \text {if } V_0 \ne \frac{2(q_3-q_1)}{g + q_3-q_1}, \end{aligned}$$

where *T*(*c*) is defined by (14) and $c_\pm$ are defined by (16a)–(16b).

**Fig. 2:**
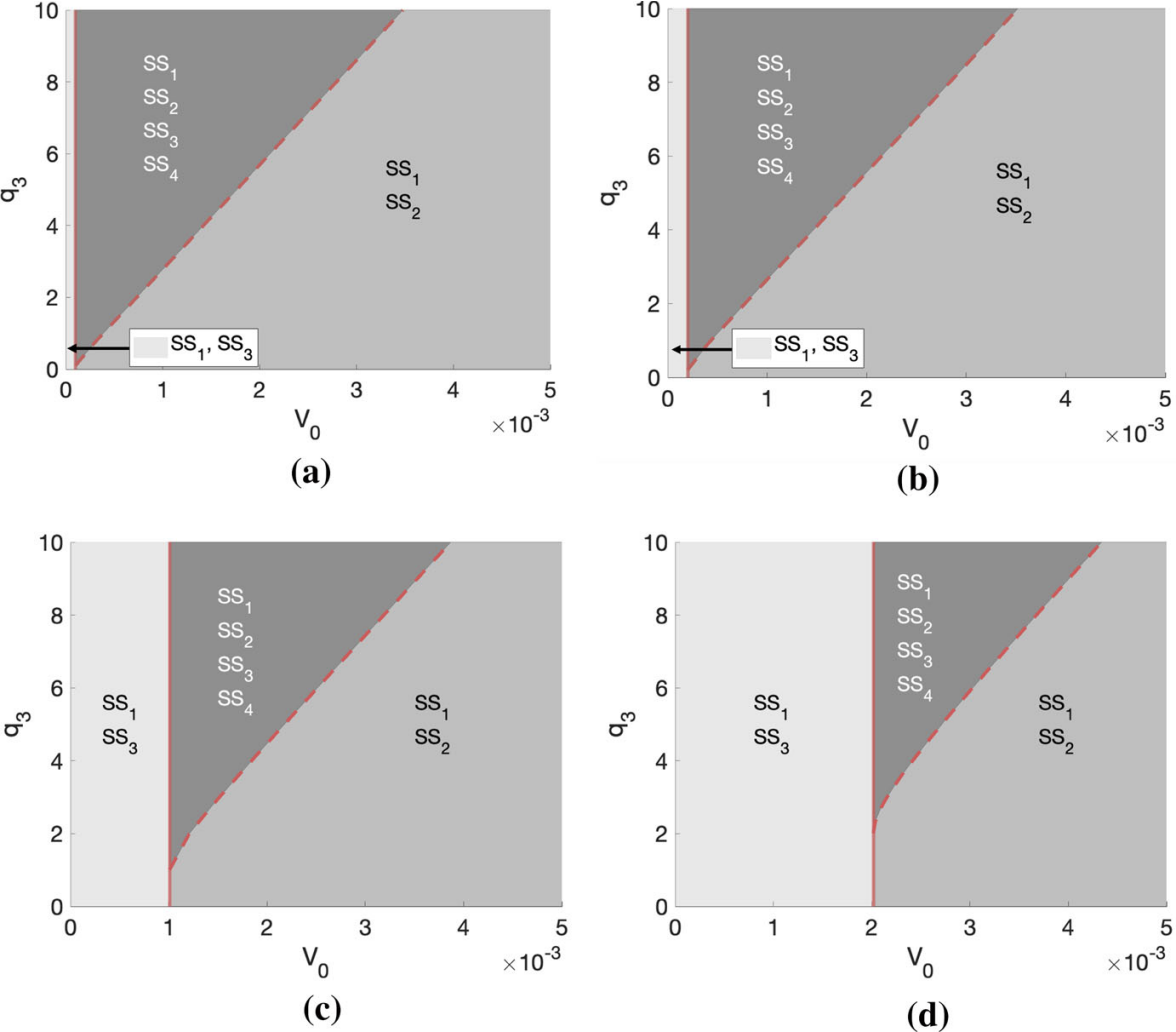
The plots (**a**)–(**d**) represent the regions of existence for the steady states $\mathrm {SS}_1$–$\mathrm {SS}_4$ of the system (4)–(5) in $(V_0,q_3)$-space for $q_1 \in \{ 0.05, 0.1, 0.5, 1\}$, respectively. The solid and dashed red lines represent the boundaries between the three distinct regions that can be observed

Similarly to the SL steady states, in order to exist and be physically realistic, $\mathrm {SS}_3$ and $\mathrm {SS}_4$ must satisfy $0 \le T \le 1-V_0$ and $0\le c < c_{\min }$. Given the expression for *T*(*c*) provided in (14) and assuming that $0 \le c < c_{\min }$, we have $T(c)< 1-V_0$ for any parameter combination. Due to the complexity of the expressions for the steady states $\mathrm {SS}_3$ and $\mathrm {SS}_4$, extensive algebraic manipulation is required to determine the regions in parameter space in which the remaining conditions hold. We therefore fix $g= 5$ and $c_{\min }=0.01$ and use Mathematica to determine the regions in $(V_0,q_3)$-space for different fixed values of $q_1$ in which $\mathrm {SS}_3$ and $\mathrm {SS}_4$ exist as admissible steady-state solutions. We illustrate these regions in Fig. 2 and we also include the regions in which $\mathrm {SS}_1$ and $\mathrm {SS}_2$ are admissible, as defined previously.

##### Remark 2

Figure 2 corresponds to specific values of *g* and $c_{\min }$. We can, however, obtain qualitatively similar results for different values of these parameters. In particular, for $q_1$ fixed, increasing *g* shifts the regions of existence of each steady state to the left, which decreases the size of the regions in which both $\mathrm {SS}_1$ and $\mathrm {SS}_3$ and all steady states exist and increases the size of the region in which both $\mathrm {SS}_1$ and $\mathrm {SS}_2$ exist. In contrast, for $q_1$ fixed, increasing $c_{\min }$ shifts the regions of existence of each steady state to the right, which increases the size of the regions in which both $\mathrm {SS}_1$ and $\mathrm {SS}_3$ and all steady states exist and decreases the size of the region in which both $\mathrm {SS}_1$ and $\mathrm {SS}_2$ exist.

Steady states $\mathrm {SS}_3$ and $\mathrm {SS}_4$, which always satisfy $\mathrm {SS}_3 < \mathrm {SS}_4$, represent tumours that are not SL (i.e. $0< T(c) < 1 - V_0$); their growth is limited by nutrient availability, and at steady state, the rates of cell proliferation and death are nonzero and balance. From Fig. 2, we see that, for fixed *g* and $c_{\min }$, $\mathrm {SS}_3$ exists when (i) there is no non-trivial SL steady state, i.e. $V_0$ cannot sustain a SL tumour’s oxygen requirements for maintenance or (ii) there is a non-trivial SL steady state, i.e. $V_0$ can sustain a SL tumour’s oxygen requirements for maintenance, but the oxygen requirements for proliferation ($q_3$) are high. In contrast, for fixed *g* and $c_{\min }$, $\mathrm {SS}_4$ only exists in case (ii).

#### Stability Analysis

In the previous section, we defined the model’s steady-state values and their regions of existence in $(V_0,q_1,q_3)$-space for fixed *g* and $c_{\min }$. We now investigate their stability.

*Spatially Limited Steady States.* We first perform a linear stability analysis for steady states $\mathrm {SS}_1$ and $\mathrm {SS}_2$; we compute the Jacobian of the system (4)–(5) when $c_{\min } \le c \le 1$ at each steady state and find the corresponding eigenvalues. The Jacobians of the system evaluated at $\mathrm {SS}_1$ and $\mathrm {SS}_2$ are, respectively:2021It is straightforward to obtain the eigenvalues $(\lambda _1,\lambda _2)$ and $(\mu _1,\mu _2)$ for the Jacobians evaluated at $\mathrm {SS}_1$ and $\mathrm {SS}_2$, respectively:

22 $$\begin{aligned} {\left\{ \begin{array}{ll} \displaystyle \lambda _1 = q_2(1-V_0) > 0, \\ \displaystyle \lambda _2 = -gV_0< 0, \end{array}\right. } \quad {\left\{ \begin{array}{ll} \displaystyle \mu _1 = \frac{-q_2(1-V_0)V_0}{V_0 + (q_1/g) (1-V_0)}< 0, \\ \displaystyle \mu _2 = -gV_0-q_1(1-V_0) < 0. \end{array}\right. } \end{aligned}$$

Since $q_1, q_2>0$ and $0<V_0<1$, it is clear that steady state $\mathrm {SS}_1$ is unstable and steady state $\mathrm {SS}_2$ is stable. Thus, in the SL regime, tumour elimination is not possible and the tumour persists.

*Nutrient-Limited Steady States.* We can perform a similar stability analysis for $\mathrm {SS}_3$ and $\mathrm {SS}_4$. The Jacobian of the system (4)–(5) when $0 \le c < c_{\min }$ is:

23 $$\begin{aligned} J |_{(T^*,c^*)}&= \begin{pmatrix} q_2c^*(1-2T^*-V_0)-\delta _1(c_{\min }-c^*) &{} q_2T^*(1-T^*-V_0)+\delta _1 T^* \\ -q_1 - q_3(1-2T^*-V_0) &{} -gV_0-q_1T^*-q_3T^*(1-T^*-V_0) \end{pmatrix}.\quad \quad \end{aligned}$$

**Fig. 3:**
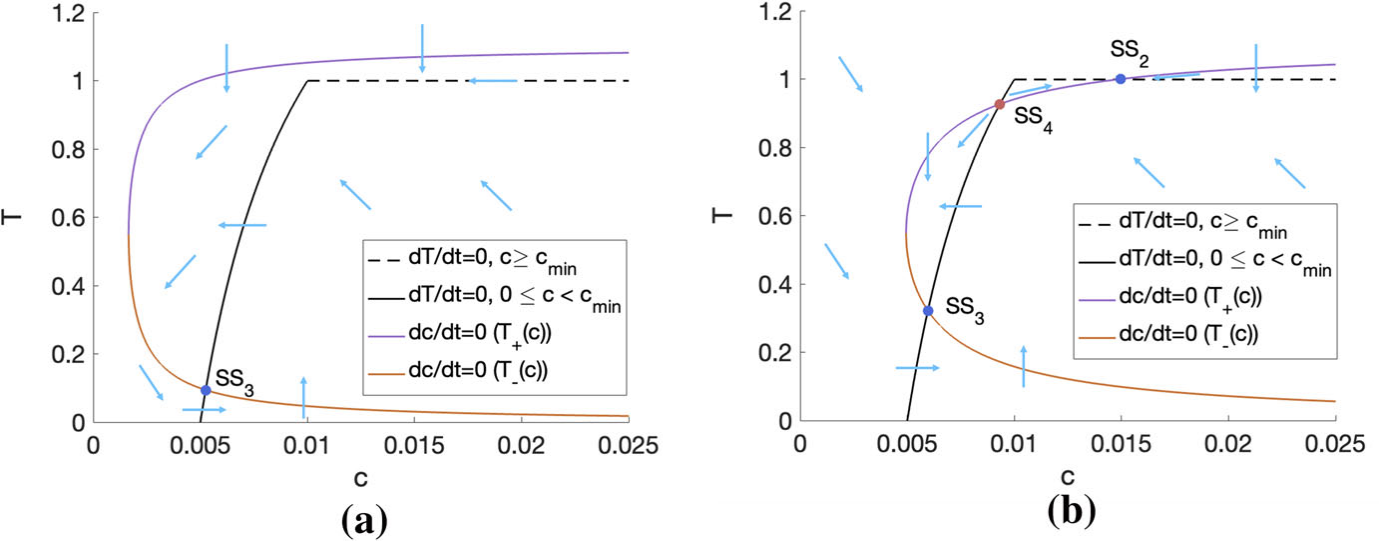
Phase portrait for Eqs. (4)–(5), where (**a**) $(V_0,q_1,q_3) = (0.0005, 0.5, 5)$ and (**b**) $(V_0,q_3,q_1) = (0.0015, 0.5, 5)$. From (**a**) and (**b**), we can find the sign of the component $J_{21}$ of the Jacobian (23) evaluated at $\mathrm {SS}_3$ and $\mathrm {SS}_4$: $J_{21}$ is, respectively, negative and positive. Since, for $\mathrm {SS}_3$, we also have $J_{11}$, $J_{22} < 0$ and $J_{12} > 0$, this implies that $\mathrm {SS}_3$ is stable. In contrast, we cannot definitively determine the stability of $\mathrm {SS}_4$ using the signs of the components of the Jacobian. However, we can see from the trajectories in **b** that $\mathrm {SS}_4$ is unstable

Using the fact that Eqs. (12a)–(12b) hold at both steady states $\mathrm {SS}_3$ and $\mathrm {SS}_4$, it is straightforward to show that $J_{11} < 0$, $J_{22} < 0$ and $J_{12} > 0$ for $J |_{\mathrm {SS}_3}$ and $J |_{\mathrm {SS}_4}$ (for all possible parameter sets). However, determining the sign of $J_{21}$ for either $\mathrm {SS}_3$ or $\mathrm {SS}_4$ is complicated given the algebraic complexity of the expressions of *T* and *c* for these steady states. Therefore, we establish the sign of $J_{21}$ in specific cases by performing phase plane analyses. Figure 3 contains two phase portraits for sets of parameters that, respectively, correspond to a region of parameter space where the only NL steady state is $\mathrm {SS}_3$ and where the two NL steady states exist (see Fig. 2). We can deduce from these phase portraits that, for these parameter sets, $J_{21}<0$ for $\mathrm {SS}_3$, whereas $J_{21}>0$ for $\mathrm {SS}_4$. Phase portraits for other parameter sets (not shown) suggest that the sign of $J_{21}$ remains the same for both steady states, irrespective of the values of the parameters.

Given the signs of each component of the Jacobian evaluated, respectively, at $\mathrm {SS}_3$ and $\mathrm {SS}_4$, we now discuss the stability of these two steady states. First of all, since $J_{11} < 0$ and $J_{22} < 0$ for $\mathrm {SS}_3$ and $\mathrm {SS}_4$, the trace of the Jacobian is negative for both steady states. For $\mathrm {SS}_3$, the determinant of the Jacobian is positive, which implies that $\mathrm {SS}_3$ is stable. In contrast, for $\mathrm {SS}_4$, the determinant could be positive or negative depending on the parameter values (since $J_{11} J_{22} > 0$ and $J_{12} J_{21} > 0$). We therefore cannot confirm whether $\mathrm {SS}_4$ is stable or not, but the phase portrait in Fig. 3b shows that $\mathrm {SS}_4$ is unstable for that specific parameter set. Constructing phase portraits for other parameter sets (not shown) suggests that $\mathrm {SS}_4$ is unstable, irrespective of parameter values. We therefore conclude that $\mathrm {SS}_3$ is stable, and make the conjecture that $\mathrm {SS}_4$ is unstable.

### The Existence of Three Tumour Growth Regimes

**Fig. 4:**
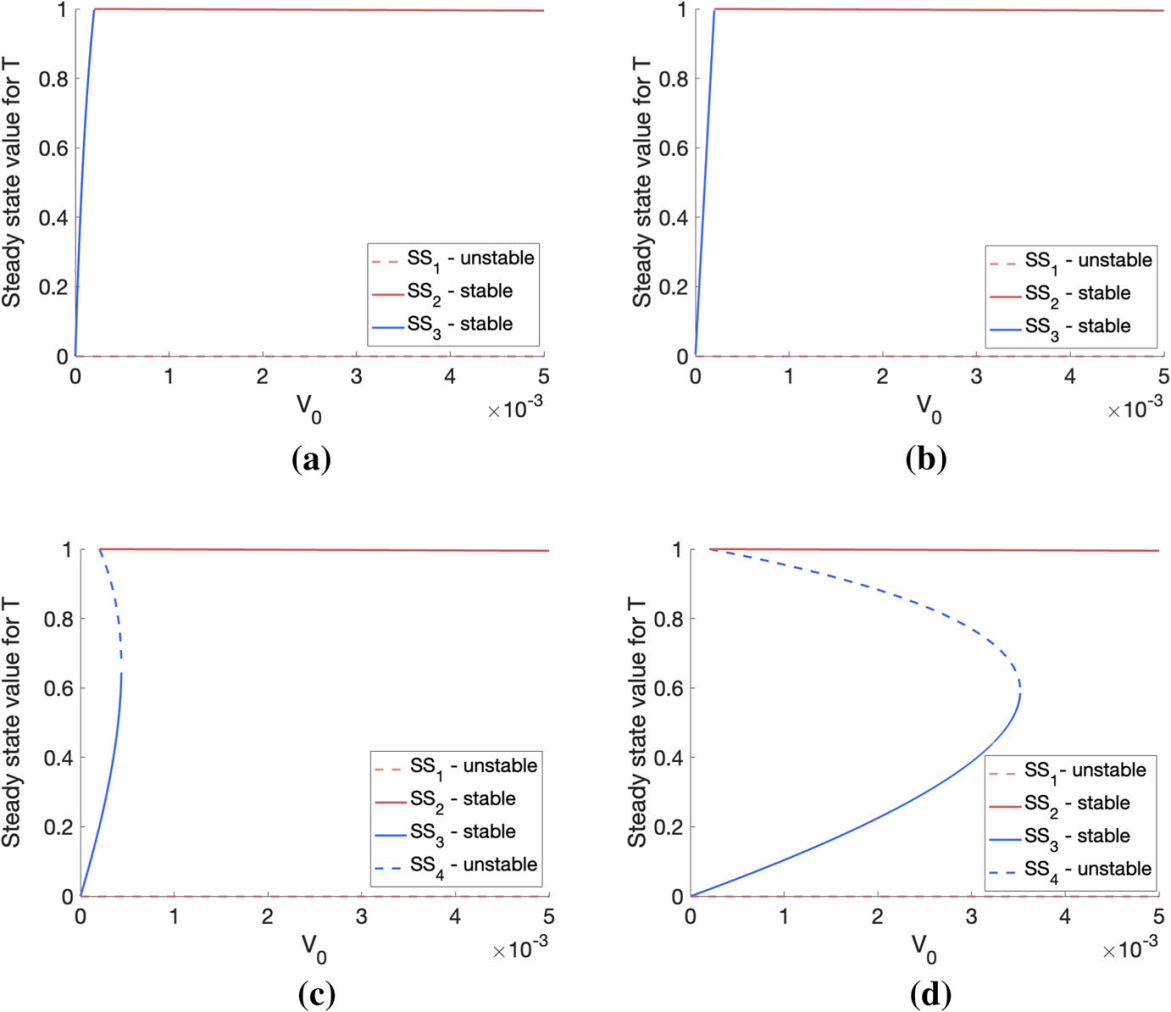
Bifurcation diagrams showing how the steady-state values for *T* change as $V_0$ varies for (**a**) $(q_1,q_3)= (0.1, 0.01)$, (**b**) $(q_1,q_3)= (0.1, 0.1)$, (**c**) $(q_1,q_3)= (0.1, 1)$ and (**d**) $(q_1,q_3)= (0.1, 10)$. We observe that, given $q_1$ sufficiently small (here, $q_1=0.1$), the existence of the bi-stable regime depends on $q_3$ being sufficiently large relative to $q_1$

In the two previous sections, we defined the steady-state solutions for Eqs. (4)–(5), determining where they exist in parameter space, and we also found their respective stabilities. These results enable us to understand how the choice of parameters influences the equilibrium tumour size(s) that can be attained in the long term. To illustrate this, we constructed bifurcation diagrams which show how the stable and unstable steady-state solutions for *T* change as $V_0$ varies, for fixed values of $q_1$ and $q_3$. These are presented in Fig. 4.

We see that, if $q_3$ is smaller than $q_1=0.1$, then the system evolves to a SL or NL steady state and there is no bi-stability. In contrast, if $q_3$ is sufficiently large relative to $q_1=0.1$, then a bi-stable region exists in addition to those mono-stable regions in which *T* automatically attains either a SL or a NL steady state. As $q_3$ increases, the region of bi-stability increases in size. This is consistent with Fig. 2, which presents the regions of existence of each steady state and also shows that bi-stability only occurs if $q_1$ is sufficiently small relative to $q_3$.

**Fig. 5:**
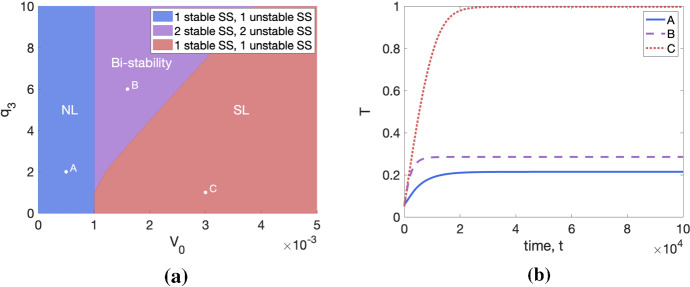
In (**a**), we represent the three tumour growth regimes in $(V_0,q_3)$-space for $q_1=0.1$. In (**b**), we numerically solve the system (4)–(5) for $t\in (0,10^5]$ subject to the initial conditions $(T(0),c(0))=(0.05,1)$ and plot the evolution of the tumour volume in time. We set $(V_0,q_1,q_3)$ corresponding to points A, B and C in (**a**), i.e. $(V_0,q_3,q_1)$ = (0.005, 0.15, 0.1), $(V_0,q_3,q_1)=(0.014, 0.8, 0.1)$ and $(V_0,q_3,q_1)=(0.035,0.5,0.1)$, respectively. We observe that a tumour characterised by parameter set A grows to a SL steady state, while the tumours characterised by parameter sets B and C both grow to a NL steady state

Combining all of the preceding results, we conclude that, depending on $(V_0,q_1,q_3)$, there are three possible scenarios for tumour growth, which we illustrate in Fig. 5:

1. *Spatially limited growth*: the tumour has access to sufficient nutrient to keep growing until it runs out of physical space to occupy; growth stops due to density-dependent inhibition which stops cell proliferation without any cell death.
2. *Nutrient-limited growth*: the tumour grows until the birth of new cells balances the death of cells due to nutrient starvation; growth stops due to nutrient-dependent inhibition.
3. *Bi-stability*: the system is bi-stable and the tumour can grow to a NL or SL steady state, depending on the initial conditions. By considering trajectories in the phase plane, it is possible to show that, given initial conditions that satisfy $0 < T(0) \ll 1$, the tumour will grow to a NL steady state (for example, see Fig. 5b).

## Discussion

Understanding tumour growth dynamics is essential for investigating tumour response to treatment. This is why developing tumour growth models and extending them to study treatment response has been at the forefront of mathematical oncology research for many years. As increasing numbers of models are proposed, a key question is how to balance the biological detail of a model with its analytical tractability and suitability for data fitting and parameter estimation. We have attempted to strike that balance by proposing an ODE model of vascular tumour growth which retains the simplicity of phenomenological models while incorporating some mechanistic details. In addition, a key goal was to develop a model that incorporates two alternative mechanisms for tumour growth arrest.

By studying the behaviour of the model (4)–(5) numerically, we found that the desired qualitative growth trends are preserved: we observed exponential and/or linear growth, followed by convergence to a parameter-dependent limiting size. Our steady-state analysis further revealed that, depending on the oxygen consumption rates and the initial vascular volume, the model exhibits three distinct tumour growth regimes. These regimes are characterised by two different growth-limiting processes: growth arrest due to cell proliferation balancing cell death due to nutrient starvation vs. growth arrest without cell death where cells stop proliferating due to space constraints.

Despite the simplicity of the model, we expect that it can provide additional insight compared to other models of tumour growth, which typically only represent a single growth-limiting mechanism (Drasdo and Höhme 2005; Greenspan 1972; Hahnfeldt et al. 1999; Lewin et al. 2020; Murphy et al. 2016; Panovska et al. 2007). This is because the mechanisms responsible for growth arrest may influence how a tumour responds to a particular treatment. We illustrate this below with two simple examples that motivate how our model can be used to study tumour response to various treatments. We first consider a treatment, here called treatment 1, that causes oxygen-independent vascular damage and damage-induced angiogenesis (e.g. high hyperthermia). In particular, this treatment can significantly alter the vascular volume, *V*, which was held constant in the tumour growth model presented in this paper. Since the tumour steady state depends on the oxygen consumption rates, $$q_3$$ and $$ q_1$$, and the vascular volume, $$V \equiv V_0$$, changes to the vascular volume will affect the steady-state tumour volume. This may, in turn, also change the tumour’s growth regime (i.e. nutrient-limited (NL), bi-stable or spatially limited (SL)) and the mechanism driving growth arrest.

**Fig. 6:**
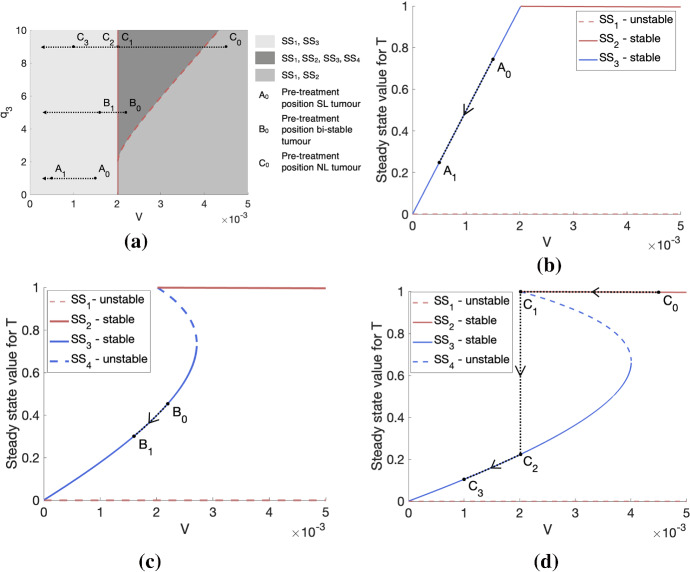
We illustrate how tumours that belong to different growth regimes respond to treatment 1, under the assumption that the vascular volume, *V*, is a monotonically decreasing function of the dose, *D*, of treatment 1. In (**a**), we show how a nutrient-limited (NL) tumour, a tumour in a bi-stable regime and a spatially limited (SL) tumour, respectively characterised by the parameters $(V_0,q_1,q_3)=(0.0022,1,1)$, $(V_0,q_1,q_3)=(0.0022,1,5)$ and $(V_0,q_1,q_3)=(0.0045,1,9)$, traverse the parameter space as *V* decreases in response to the application of increasing doses of treatment 1. $A_0$, $B_0$ and $C_0$ respectively represent the pre-treatment position of these three tumours in parameter space. In (**b**)–(**d**), we respectively show, using bifurcation diagrams, how the steady-state volumes of these three tumours change in response to the same treatment. We see that, for the tumours initially in NL (**b**) and bi-stable (**c**) regimes, their steady-state volumes both decrease gradually with *V*. For the tumour initially in a SL regime (**d**), decreasing *V* initially leads to a slight increase in tumour steady-state volume. However, a sufficiently large decrease in *V* can cause a large and rapid reduction in tumour steady-state volume that is followed by a continued, gradual decrease

To illustrate this, let us assume that *V* is a monotonically decreasing function of the dose *D* of treatment 1. Then, as *D* increases, the value of *V* in the model Eqs. (4)–(5) changes so that the tumour traverses regions of parameter space in ways that depend on the initial vascular volume, $V_0$, as well as other model parameters (see Fig. 6a). This can result in the system moving through different sequences of steady-state behaviour. We demonstrate this using the bifurcation diagrams in Fig. 6, which show how the tumour steady-state volume depends on the vascular volume, for fixed $(q_3,q_1)$. On the one hand, tumours initially in NL or bi-stable regimes exhibit an immediate and gradual decrease in tumour steady-state volume as *V* decreases (Fig. 6b, $A_0$ to $A_1$, and Fig. 6c, $B_0$ to $B_1$). In these cases, the steady-state solutions remain on the NL steady-state branch of the bifurcation diagrams in Fig. 6b, c as *V* decreases. On the other hand, the steady-state size of a tumour initially in a SL regime increases marginally as *V* decreases towards a threshold value (Fig. 6d, $C_0$ to $C_1$). Once this value is reached, the tumour undergoes a rapid and large reduction in steady-state volume as it switches to the NL regime (Fig. 6d, $C_1$ to $C_2$). As *V* continues to decrease, the tumour undergoes a more gradual, sustained decrease in steady-state volume, remaining in the NL regime (Fig. 6d, $C_2$ to $C_3$) and behaving similarly to tumours that are initially in bi-stable or NL regimes. In this case, the steady-state solution can jump from the SL steady-state branch to the NL steady-state branch of the bifurcation diagram in Fig. 6d, provided that treatment 1 elicits a sufficiently large decrease in *V*.

These possible variations of tumour steady-state values and growth regimes in response to treatment 1 highlight how such a treatment can be effective at reducing long-term tumour burden, especially in the case of SL tumours. In particular, the preceding analysis emphasises that treatment of SL tumours should be designed to drive the system into the NL regime, where the larger spatially limited tumour steady state does not exist and the tumour is guaranteed to evolve to a reduced volume. It would therefore be interesting to consider the effect of combining treatment 1 with another treatment such as chemotherapy that, say, causes tumour cell damage. However, elucidating tumour response to such a combined treatment would require us to consider the dynamics of the tumour cells and vasculature and their interactions. In particular, we would need to extend our model to include a further ODE to account for the dynamics of *V*: this is beyond the scope of the present work. Even so, we can illustrate the potential of our current model for studying combination treatments using the following example.

**Fig. 7:**
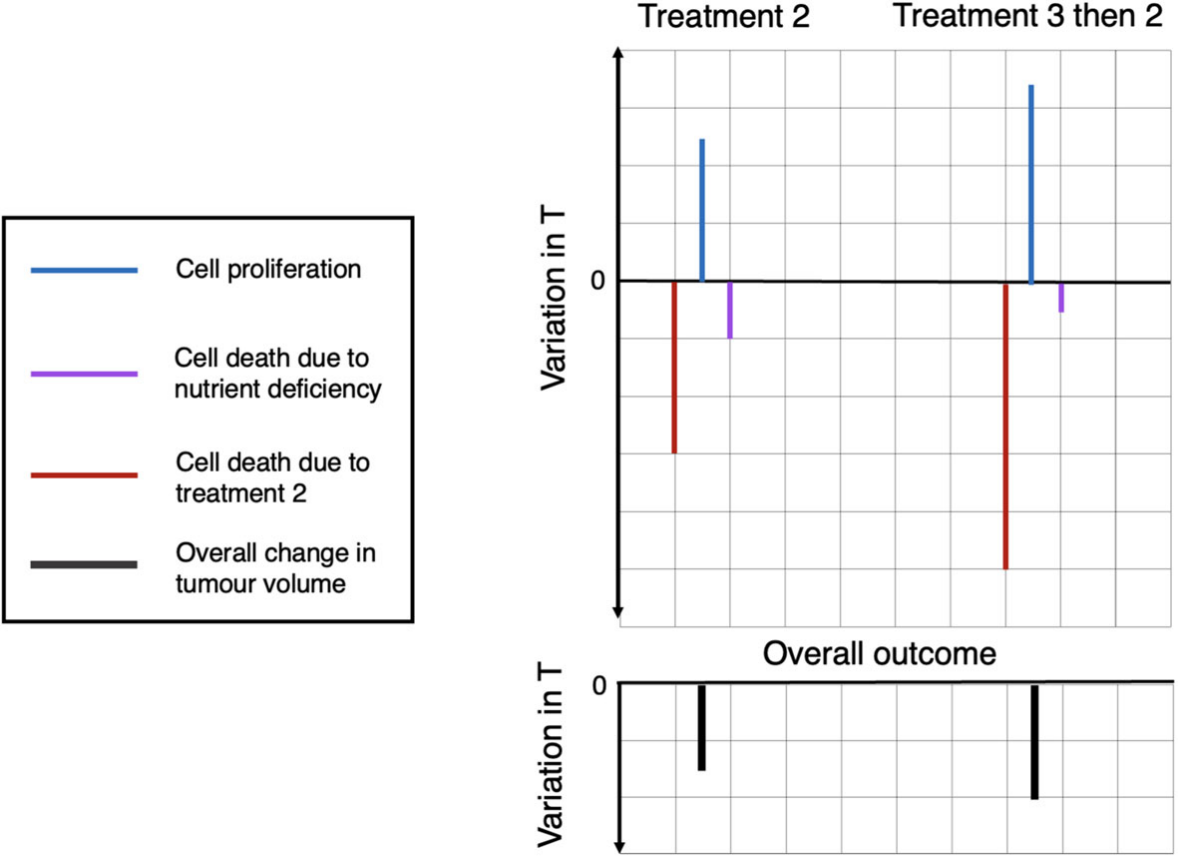
Schematic diagram showing a possible outcome of combining treatment 2, which causes oxygen-dependent cell damage, with treatment 3, which re-oxygenates the tumour, affects cell proliferation, cell death due to nutrient deprivation and cell kill due to treatment 2. The combination of these effects determines the overall outcome of treatment. In the case considered here, the magnitude of the effects of treatment 3 on the individual aspects of growth and death are such that we observe an increase in tumour cell death as a result of combining the two treatments. However, as shown in Fig. 8, this may not always be the case

Suppose that we combine a treatment, here called treatment 2, which causes tumour cell damage in an oxygen-dependent manner (e.g. radiotherapy) with another treatment, here called treatment 3, which can re-oxygenate the tumour (e.g. mild hyperthermia). Treatment 2 alone causes a decrease in tumour volume, whose magnitude depends on how cell death due to the treatment and nutrient deficiency compares to cell proliferation. By increasing the oxygen concentration in the tumour, treatment 3 has three key effects: (i) increasing the cell kill caused by treatment 2, (ii) increasing the proliferation rate of tumour cells and (iii) decreasing the rate of cell death due to nutrient deficiency (if the death rate is nonzero). Thus, applying treatment 3 before treatment 2, we predict a synergistic benefit of combining the treatments if the magnitude of the decrease in tumour volume is larger than that achieved by treatment 2 alone. We illustrate this scenario in Fig. 7.

**Fig. 8:**
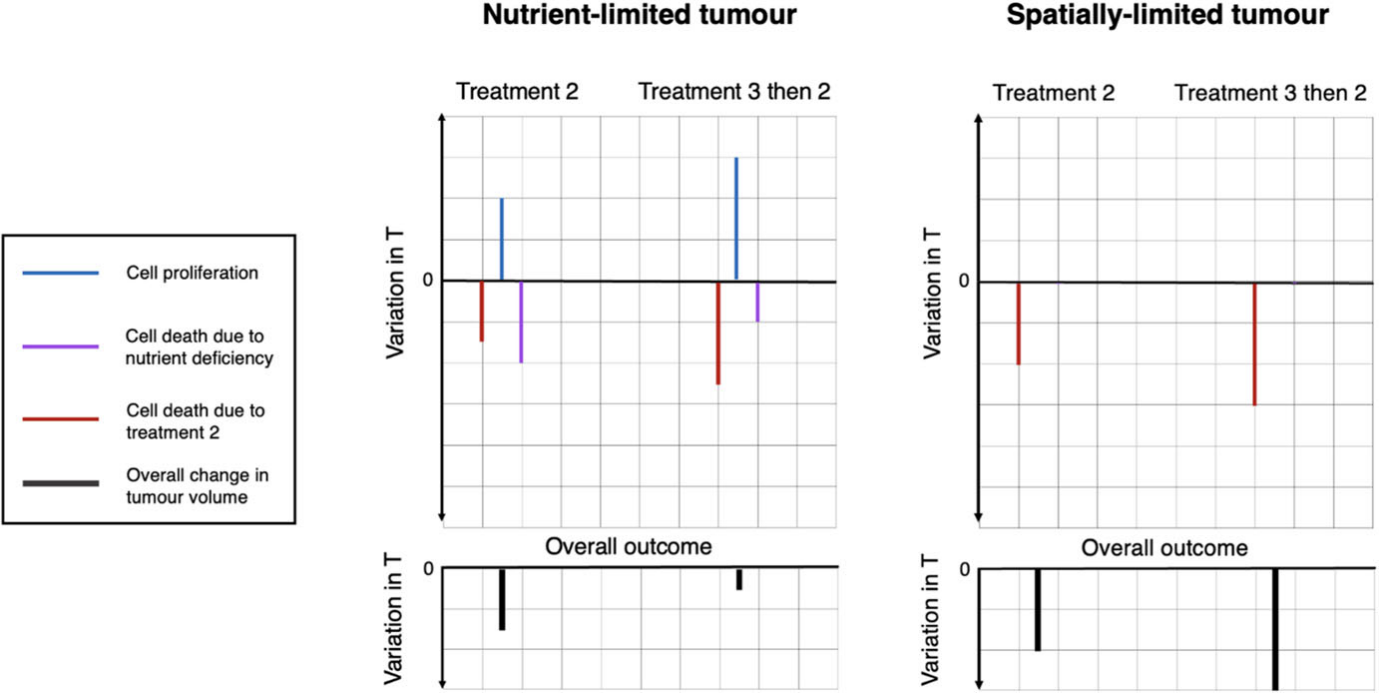
We compare the response of a steady-state NL and a steady-state SL tumour to treatment 2 alone and to a combination of treatment 3 followed by treatment 2. The two schematic diagrams show how combining treatment 2, which causes oxygen-dependent cell damage, with treatment 3, which re-oxygenates the tumour, affects cell proliferation, cell death due to nutrient deprivation and cell kill due to treatment 2. The combination of these effects then determines the overall outcome of treatment. In the case of the NL tumour, we observe a decrease in tumour cell death as a result of combining the two treatments, whereas, for the SL tumour, we observe an increase in tumour cell death as a result of combining the two treatments. For the cases considered here, the combination treatment is therefore only beneficial in the case of the SL tumour

Given the effects of re-oxygenation previously described, we claim that NL tumours are more likely to respond poorly to this combination treatment than SL tumours. Suppose that we have two tumours at steady state, one NL and one SL. Recall that, at equilibrium, the rates of cell proliferation and cell death balance for NL tumours and nutrient availability is growth rate limiting, whereas for SL tumours equilibrium is achieved when mechanical constraints halt cell proliferation throughout the entire tumour mass, i.e. nutrient is not limiting and there is no cell death. Re-oxygenation (treatment 3) perturbs the NL steady state, but not the SL steady state. More specifically, re-oxygenation allows cell proliferation to outweigh cell death due to nutrient deficiency, and therefore, the NL tumour can grow past its pre-treatment volume before the cell-damaging treatment (treatment 2) is applied. As a result of this additional tumour growth due to treatment 3, the increase in cell kill by treatment 2 due to re-oxygenation may not be sufficient to enhance the tumour’s response compared to treatment 2 alone. Thus, the combination treatment may fail. In contrast, re-oxygenating the SL tumour increases the average oxygen concentration in the tumour without affecting the rates of cell proliferation and cell death as they both remain zero. By doing so, treatment 3 only increases cell kill by treatment 2, which increases the efficacy of treatment 2 and ensures that there is a benefit of combining the treatments. We illustrate the expected responses of steady-state NL and SL tumours to treatment 2 alone and to a combination of treatment 3 followed by treatment 2 in Fig. 8.

Treating tumours that are not at steady state, we also expect to observe a percentage increase in tumour growth due to re-oxygenation that is larger in NL than SL tumours: while the growth rates of NL and SL tumours both increase due to enhanced proliferation, that of NL tumours is further increased by a decrease in cell loss. As previously explained for NL tumours at steady state, a sufficiently large increase in tumour growth following treatment 3 can negatively impact the success of the combined treatment. All of the preceding considerations imply that, by using the tumour growth model presented in this paper to study tumour response to this combination treatment, we can expect varying treatment outcomes depending on tumour type and growth-limiting mechanism.

As mentioned in the Introduction, our simple model exhibits complex behaviour that lends itself to investigating and distinguishing between the response of different tumours to a range of combination cancer therapies. While we have illustrated this briefly with the two preceding hypothetical scenarios, a detailed investigation into how this model can be exploited to address specific combination treatments will be presented in a future paper. More specifically, we aim to extend the tumour growth model to incorporate tumour response to different treatments and then use the resulting dynamic model to conduct an in-depth study of treatment outcome. In particular, we seek to explore whether the response to treatment is sensitive to the mechanisms underpinning tumour growth and to the form of tumour growth assumed. If so, we may also be able to discern the underlying mechanisms from observed responses to treatment. Moreover, our simplifying assumption that vascularisation is constant allowed us to analyse the system in depth and find behaviours that depend critically on the level of vascularisation. Hence, future work will incorporate more realistic vascular dynamics. The extensions of the study presented here will help to identify optimal, patient-specific treatment combinations and to increase our understanding of high variability in treatment response between patients.

## Supplementary Information

Below is the link to the electronic supplementary material.

Supplementary file 1 (pdf 29 KB)

## Appendix A: Defining the Parameter Regime of Interest

Here we justify our dimensionless parameter choices, which are summarised in Table 1, by referring to dimensional parameter values.

We first define a range of values for the vascular volume $V_0$. Assuming that the average diameter of a cell is $20 \,{\upmu }\hbox {m}$, we estimate that the maximum number of cells that can occupy $1 \, \hbox { mm}^{3}$ of space is $1.25 \times 10^5$ (Ardaseva 2020). Now, the Krogh cylinder model shows that oxygen can diffuse up to $100-200 \, {\upmu }\hbox {m}$. Using the average cell size and cylindrical geometry, we estimate that a section of blood vessel of length $20 \,{\upmu }\hbox {m}$ can supply oxygen to a number of cells in the range [100, 300]. In particular, given that the average diameter of a capillary is $8 \, {\upmu }\hbox {m}$ (Müller et al. 2008) and using cylindrical geometry once again, we further estimate that $1 \times 10^{-6} \, \hbox { mm}^{3}$ of blood vessel can provide oxygen for a volume of cells in the range $[8 \times 10^{-4}, 2.4 \times 10^{-3}] \, \hbox { mm}^{3}$. Therefore, a biologically realistic range for $V_0$ such that the tumour may be sufficiently oxygenated is $[10^{-4},5 \times 10^{-3}]$. Since tumour vasculature is usually less effective than healthy vasculature (Carmeliet and Jain 2000), we consider $V_0 \in (0, 5 \times 10^{-3}]$.

We now determine $c_{\min }$, *k* and *g*. To do so, we first estimate $S_{\max } = 10^{-6} \, \hbox { m}^{3}$ in accordance with the average size of vascular tumours in mice (Faustino-Rocha et al. 2013; Wu et al. 2013). Moreover, the average oxygen partial pressure in peripheral normal tissues is approximately $p_{\max } = 38 \,\hbox { mmHg}$ (Ortiz-Prado et al. 2019), and therefore, using Henry’s Law to convert $p_{\max }$ into a concentration, it is straightforward to calculate $c_{\max }=2.1 \times 10^{-3} \, \hbox { kg} \hbox { m}^{-3}$. Now, hypoxia is attained when oxygen levels fall below $8\, \hbox { mmHg}$ (McKeown 2014). Unlike normal tissue cells, tumour cells can survive in such hypoxic conditions and only start to die in severely hypoxic conditions, which range from $0.75\, \hbox { mmHg}$ to $0.075\, \hbox { mmHg}$ depending on the tumour (McKeown 2014). Hence, we take the average of these two values, and using Henry’s Law again, we have $c^*_{\min } = 2.26 \times 10^{-5} \, \hbox { kg} \hbox { m}^{-3}.$ This implies that

A1 $$\begin{aligned} c_{\min } = \frac{c^*_{\min }}{c^*_{\max }} = \frac{2.26}{2.1} \times 10^{-2} \approx 10^{-2}, \end{aligned}$$

and we therefore set $c_{\min } = 10^{-2}$.

In addition, we expect that the rate of oxygen exchange per unit volume area of blood vessels, $g^*$ (${\hbox { m}^{-3} {\min }^{-1}}$), depends on a variety of factors, including the quality of the vascular network, which can lead to highly heterogeneous tumour perfusion rates (Gillies et al. 1999), and surface areas of oxygen exchange. Since our model does not account for these details, we fix $g^*$ so that, for average values of the oxygen consumption rates, we have SL tumours for $V_0 \ge 5 \times 10^{-3}$ and NL or bi-stable tumours for $V_0 \le 1 \times 10^{-4}$ (recall that, for $V_0 \in [10^{-4}, 5 \times 10^{-3}]$, the vasculature should be sufficient to supply oxygen to the whole tumour). Based on preliminary numerical simulations (not shown), we set $g^* = 5 \times 10^{6} \, {\hbox { m}^{-3} {\min }^{-1}}$. Setting the timescale to be $\tau = 1 \, \hbox { min}^{-1}$, we thus have

A2 $$\begin{aligned} g = \frac{g^* S_{\max }}{\tau } = 5. \end{aligned}$$

Finally, $k^*$ ($\mathrm {m}^6$ $\hbox { kg}^{-1}$) is the parameter that relates the constant for oxygen consumption for proliferation, $q^*_3$, and the constant for proliferation, $q^*_2$. Since cells consume oxygen faster than they proliferate, we expect $q^*_2 < q^*_3$. As the value of $k^*$ is not readily available in the literature, we performed preliminary numerical simulations (not shown) to find a value of $k^*$ that gives realistic growth dynamics. As a result, we fix $k^* = \frac{10^{-5}}{2.1}$ $\mathrm {m}^6 {\hbox { kg}^{-1}}$ and this implies that

A3 $$\begin{aligned} k = \frac{c^*_{\max }}{S_{\max }} k^* = \frac{2.1 \times 10^{-3} \times 10^{-5} \, \hbox { kg} \hbox { m}^{-3} \mathrm {m}^6 {\hbox { kg}^{-1}} }{2.1 \times 10^{-6} \hbox { m}^{3} } = 10^{-2}. \end{aligned}$$

We now estimate the oxygen consumption rates, the proliferation rate and the death rate. Biological observations suggest that the death and proliferation rates of tumour cells are highly correlated in some tumours: the faster the growth, the larger the death rate (Leoncini et al. 1993; Liu et al. 2001; Vaquero et al. 2004). This suggests that these rates are proportional to each other and we make the simplifying assumption that the dimensionless parameters $\delta _1$ and $q_2$ satisfy $\delta _1 = q_2$ in order to reduce the number of parameters in the model. We note here that this assumption does not affect the qualitative behaviour of the model summarised in the paper, and in particular, the three tumour growth regimes are preserved.

Now, we estimate $\delta ^*_1$ (${ \hbox { m}^{3} \hbox { kg}^{-1}\hbox { min}^{-1}}$) as

A4 $$\begin{aligned} \delta _1^* \in \left( \frac{1.2 \times 10^{-6} \, }{0.75 \times 2.1 \times 10^{-5}}, \frac{1.2 \times 10^{-4}\,}{0.75 \times 2.1 \times 10^{-5}} \right) \frac{{\text {min}}^{-1}}{{\text {kg\,m}}^{-3}}, \end{aligned}$$

where the numerators of both fractions correspond to death rates found in the literature (Lewin et al. 2018; Schaller and Meyer-Hermann 2006) and the denominator corresponds to the average oxygen concentration below $c_{\min }$ (this scaling allows us to obtain the correct units for $\delta _1^*$). We then have:

A5 $$\begin{aligned} \delta _1^* \in \left( 7.6 \times 10^{-2}, 7.6\right) {\text {m}}^{3}\, {\text {kg}}^{-1}\,{\text {min}}^{-1}, \end{aligned}$$

which we non-dimensionalise according to (6) to obtain:

A6 $$\begin{aligned} \delta _1 \in (1.6 \times 10^{-4}, 1.6 \times 10^{-2}). \end{aligned}$$

Now, since $q_2=\delta _1$ by assumption, we have

A7 $$\begin{aligned} q_2 \in (1.6 \times 10^{-4}, 1.6 \times 10^{-2}), \end{aligned}$$

and this gives us the following dimensionless ranges for $q_3 = q_2/k$:

A8 $$\begin{aligned} q_3 \in (1.6 \times 10^{-2}, 1.6). \end{aligned}$$

We further justify the range of values determined for $q_3$ by considering experimentally determined values for the rate of oxygen consumption by cells. Wagner et al. (2011) conduct their own experiments as well as review results from other authors and they report oxygen consumption rates in the range $[6 \times 10^{-5}, 6 \times 10^{-3}] \, {\hbox {kg}\hbox { m}^{-3} {\min }^{-1}}$. These constant consumption rates correspond to the oxygen uptake of cells in a well-oxygenated medium, i.e. where the rate of oxygen consumption is at its maximum. Given the volume of the tumours in their experiments and the maximal oxygen concentration they set, we can find the following estimate for the range of $q_1^*$:

A9 $$\begin{aligned} q_1^* \in (2.1 \times 10^4, 2.1 \times 10^7) \, {\hbox { m}^{-3} {\min }^{-1}}. \end{aligned}$$

Given (6) and $S_{\max } = 10^{-6}$, this implies that

A10 $$\begin{aligned} q_1 \in (2.1 \times 10^{-2}, 21). \end{aligned}$$

Here, we determine a range of values for $q_1$ using consumption rates that correspond to overall oxygen consumption, i.e. they do not distinguish between consumption for proliferation and for maintenance. In practice, $q_1$ would therefore be smaller than the values in (A10). In view of this, we recover the dimensionless parameter ranges in Table 1 by rounding down to the closest order of magnitude the upper and lower bounds of the parameter ranges we found. We note once more that, since this paper focuses on demonstrating the qualitative behaviour of the model (4)–(5), defining plausible ranges for parameter values is sufficient.

## Declarations

### Code availability

MATLAB code to numerically solve the model are available in the Supplementary Material.
