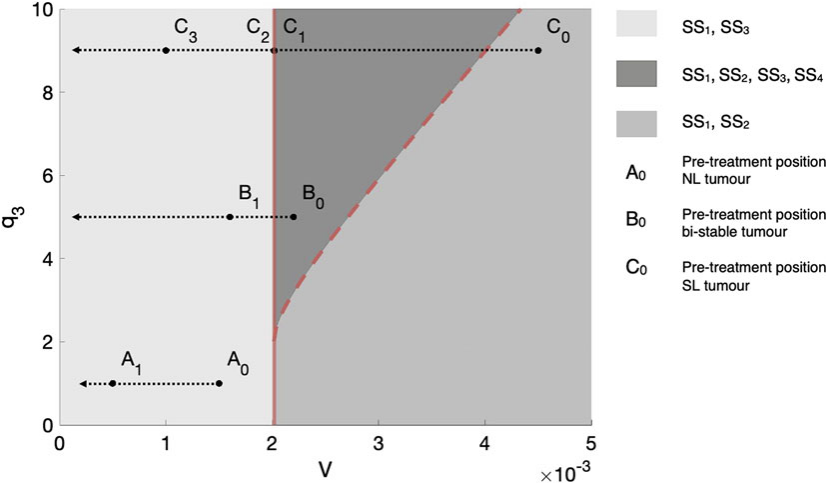# Correction to: Combining Mechanisms of Growth Arrest in Solid Tumours: A Mathematical Investigation

**DOI:** 10.1007/s11538-024-01382-1

**Published:** 2025-02-12

**Authors:** Chloé Colson, Helen M. Byrne, Philip K. Maini

**Affiliations:** Wolfson Centre for Mathematical Biology, Mathematical Institute, University of Oxford, Radcliffe Observatory Quarter, Oxford, OX2 6GG UK

**Correction to: Bulletin of Mathematical Biology** 10.1007/s11538-022-01034-2

In this article, there were minor typos in the captions of Figs. 3, 5 and 6, and in the legend of Fig. 6a. The correct version of Fig. 6a and corrected captions are given below with changes marked in bold.

**1. Figure 3:**

**Incorrect Caption**: Phase portrait for Eqs. (4)–(5), where (a) (*V*_0_, *q*_1_, *q*_3_) = (0.0005, 0.5, 5) and (b) (*V*_0_, *q*_3_, *q*_1_) = (0.0015, 0.5, 5). From (a) and (b), we can find the sign of the component *J*_21_ of the Jacobian (23) evaluated at SS_3_ and SS_4_: *J*_21_ is, respectively, negative and positive. Since, for SS_3_, we also have *J*_11_, *J*_22_ < 0 and *J*_12_ > 0, this implies that SS_3_ is stable. In contrast, we cannot definitively determine the stability of SS_4_ using the signs of the components of the Jacobian. However, we can see from the trajectories in b that SS_4_ is unstable.

**Correct Caption**: Phase portrait for Eqs. (4)–(5), where (a) (*V*_0_*, q*_1_*, q*_3_) = (0*.*0005*,* 0*.*5*,* 5) and (b) (*V*_0_*, ****q***_***1***_***, q***_***3***_) = (0*.*0015*,* 0*.*5*,* 5). From (a) and (b), we can find the sign of the component *J*_21_ of the Jacobian (23) evaluated at SS_3_ and SS_4_: *J*_21_ is, respectively, negative and positive. Since, for SS_3_, we also have *J*_11_, *J*_22_ < 0 and *J*_12_ > 0, this implies that SS_3_ is stable. In contrast, we cannot definitively determine the stability of SS_4_ using the signs of the components of the Jacobian. However, we can see from the trajectories in (b) that SS_4_ is unstable.

**2. Figure 5:**

**Incorrect Caption**: In (a), we represent the three tumour growth regimes in (*V*_0_, *q*_3_)-space for *q*_1_ = 0.1. In (b), we numerically solve the system (4)–(5) for *t* ∈ (0, 10^5^] subject to the initial conditions (*T*(0), *c*(0)) = (0.05, 1) and plot the evolution of the tumour volume in time. We set (*V*_0_, *q*_1_, *q*_3_) corresponding to points A, B and C in (a), i.e. (*V*_0_, *q*_3_, *q*_1_) = (0.005, 0.15, 0.1), (*V*_0_, *q*_3_, *q*_1_) = (0.014, 0.8, 0.1) and (*V*_0_, *q*_3_, *q*_1_) = (0.035, 0.5, 0.1), respectively. We observe that a tumour characterised by parameter set A grows to a SL steady state, while the tumours characterised by parameter sets B and C both grow to a NL steady state.

**Correct Caption**: In (a), we represent the three tumour growth regimes in (*V*_0_*, q*_3_)-space for ***q***_***1***_ ***= 0.5***. In (b), we numerically solve the system (4)–(5) for *t* ∈ (0*,* 10^5^] subject to the initial conditions (*T*(0)*, c*(0)) = (0*.*05*,* 1) and plot the evolution of the tumour volume in time. We set (*V*_0_*, q*_1_*, q*_3_) corresponding to points A, B and C in (a), i.e., **(*V***_***0***_, ***q***_***1***_, ***q***_***3***_**)** = **(0.0005, 0.5, 2)**, **(*V***_***0***_, ***q***_***1***_, ***q***_***3***_**) = (0.0016, 0.5, 6)** and **(*V***_***0***_, ***q***_***1***_, ***q***_***3***_**) = (0.003, 0.5, 1)**, respectively. We observe that a tumour characterised by parameter set **C** grows to a SL steady state, while the tumours characterized by parameter sets **A and B** both grow to a NL steady state.

**3. Figure 6:**

**Incorrect caption:** We illustrate how tumours that belong to different growth regimes respond to treatment 1, under the assumption that the vascular volume, *V*, is a monotonically decreasing function of the dose, D, of treatment 1. In (a), we show how a nutrient-limited (NL) tumour, a tumour in a bi-stable regime and a spatially limited (SL) tumour, respectively characterised by the parameters (*V*_0_, *q*_1_, *q*_3_) = (0.0022, 1, 1), (*V*_0_, *q*_1_, *q*_3_) = (0.0022, 1, 5) and (*V*_0_, *q*_1_, *q*_3_) = (0.0045, 1, 9), traverse the parameter space as V decreases in response to the application of increasing doses of treatment 1. *A*_0_, *B*_0_ and *C*_0_ respectively represent the pre-treatment position of these three tumours in parameter space. In (b)–(d), we respectively show, using bifurcation diagrams, how the steady state volumes of these three tumours change in response to the same treatment. We see that, for the tumours initially in NL (b) and bi-stable (c) regimes, their steady state volumes both decrease gradually with *V*. For the tumour initially in a SL regime (d), decreasing *V* initially leads to a slight increase in tumour steady state volume. However, a sufficiently large decrease in *V* can cause a large and rapid reduction in tumour steady state volume that is followed by a continued, gradual decrease.

**Correct caption:** We illustrate how tumours that belong to different growth regimes respond to treatment 1, under the assumption that the vascular volume, *V*, is a monotonically decreasing function of the dose, *D*, of treatment 1. In (a), we show how a nutrient-limited (NL) tumour, a tumour in a bi-stable regime and a spatially-limited (SL) tumour, respectively characterised by the parameters (*V*_0_*, q*_1_*, q*_3_) = (**0.0015,** 1*,* 1), (*V*_0_*, q*_1_*, q*_3_) = (0*.*0022*,* 1*,* 5) and (*V*_0_*, q*_1_*, q*_3_) = (0*.*0045*,* 1*,* 9), traverse the parameter space as *V* decreases in response to the application of increasing doses of treatment 1. *A*_0_, *B*_0_ and *C*_0_ respectively represent the pre-treatment position of these three tumours in parameter space. In (b)–(d), we respectively show, using bifurcation diagrams, how the steady state volumes of these three tumours change in response to the same treatment. We see that, for the tumours initially in NL (b) and bi-stable (c) regimes, their steady state volumes both decrease gradually with *V*. For the tumour initially in a SL regime (d), decreasing *V* initially leads to a slight increase in tumour steady state volume. However, a sufficiently large decrease in *V* can cause a large and rapid reduction in tumour steady state volume that is followed by a continued, gradual decrease.

**Figure 6a**

**Incorrect Figure** 
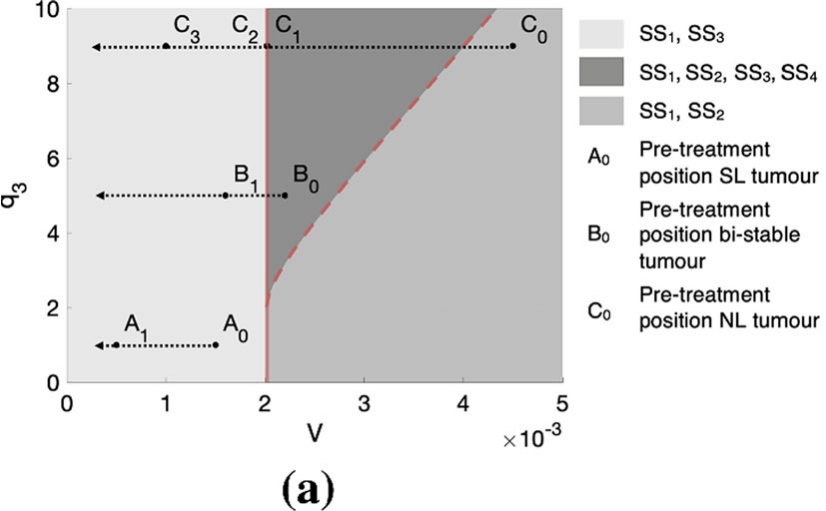


**Correct Figure**