# Supplementary material for: Combining Mechanisms of Growth Arrest in Solid Tumours: A Mathematical Investigation
Source: Bull Math Biol. 2022 Jul 1;84(8):80. doi: 10.1007/s11538-022-01034-2 (PMC9246818; doi:10.1007/s11538-022-01034-2)
Supplement: Supplementary file 1 — (pdf 29 KB) [file 11538_2022_1034_MOESM1_ESM.pdf]

```
% Code to numerically solve the ODE system using ODE45

% Defining the parameters
k = 1e-2;
c_min = 1e-2;
g = 5;
q_1 = 1;
q_3 = 5;
d_1 = k*q_3;
V_0 = 0.004;

% Defining the initial conditions
IC = [0.005, 1];

% Defining the time span
time_span = linspace(0,2.5*10^5,2.5*10^5);

% Defining the options for the ODE solver
options = odeset('AbsTol',1e-10, 'RelTol',1e-10);

% Numerically solving the system of time-dependent ODEs

[tt,uu] = ode45(@(T,U) growth(T, U, c_min, V_0, k, q_1, q_3,g,
d_1),time_span, IC, options);

% Plotting the solution for t in time_span

figure
hold on
set(gca,'FontName', 'Helvetica','FontSize',16)
plot(tt,uu(:,1),'Color',rgb('RoyalBlue'),'LineWidth',2)
plot(tt,uu(:,2),'Color',rgb('IndianRed'),'LineWidth',2)
xlabel('time, t','FontName', 'Helvetica','FontSize',16)
legend('T','c','FontName', 'Helvetica', 'FontSize',16)

% Defining the ODE system

function f = growth(t, U, c_min, V_0, k, q_1,q_3, g, d_1)
DU = zeros(2,1);


if U(2) >= c_min
    DU(1) = k*q_3*U(2)*U(1)*(1 - U(1) - V_0);
    DU(2) = (g*(1-U(2)))*V_0-q_1*U(2)*U(1) - q_3*U(2)*U(1)*(1 -  U(1) -
V_0);
elseif U(2) < c_min
    DU(1) = k*q_3*U(2)*U(1)*(1 - U(1) -V_0)-d_1*U(1)*(c_min - U(2));
    DU(2) = (g*(1-U(2)))*V_0-q_1*U(2)*U(1) - q_3*U(2)*U(1)*(1 - U(1) -
V_0);
end

f = DU;
end
```
